# Rectal metastasis from lung cancer diagnosed by endoscopic ultrasound‐guided fine needle biopsy: A case report

**DOI:** 10.1002/deo2.70127

**Published:** 2025-04-29

**Authors:** Shinji Mukawa, Yudai Koya, Tomoyuki Murakami, Koichiro Miyagawa, Yuki Maruno, Koki Yamaguchi, Shun Hanada, Shinji Oe, Masaru Harada

**Affiliations:** ^1^ Department of Gastroenterology Kyushu Rosai Hospital Moji Medical Center Fukuoka Japan; ^2^ The Third Department of Internal Medicine University of Occupational and Environmental Health Fukuoka Japan; ^3^ Department of Pathology KYURIN PACELL Corporation Fukuoka Japan

**Keywords:** endoscopic ultrasound, endoscopic ultrasound‐guided fine needle biopsy, lung cancer, metastatic colorectal cancer, rectal metastasis

## Abstract

A 73‐year‐old man visited our hospital due to hyperintestinal peristalsis and diarrhea. He had been undergoing regular annual checkups for dust lung disease. Contrast‐enhanced computed tomography scan showed edematous thickening of the rectal wall with contrast effect. A colonoscopy revealed a submucosal tumor‐like protrusion in the Rb lesion of the rectum without neoplastic epithelial changes. Forceps biopsies of the overlying mucosa were non‐diagnostic; however, endoscopic ultrasound‐guided fine needle biopsy revealed that the specimen was poorly differentiated non‐small cell invasive carcinoma. Then, we performed a chest computed tomography and a newly detected mass lesion in the upper lobe of the right lung. Based on immunohistochemical analysis and image findings, the patient was diagnosed with rectal metastasis from lung cancer. Subsequently, sputum cytology confirmed the diagnosis of lung adenocarcinoma. Rectal submucosal tumor‐like protrusions are occasionally encountered. When a non‐exposed rectal tumor is identified, it is important to differentiate metastatic diseases, consider endoscopic ultrasound‐guided fine needle biopsy, and make a definitive diagnosis through detailed immunohistochemical evaluation and systemic imaging surveillance.

## INTRODUCTION

Metastatic colorectal cancer is a rare condition. Rectal involvement is often exposed to the mucosa and is diagnosed using endoscopic forceps biopsy. However, diagnosing non‐exposed tumors is difficult. Here, we report a patient with rectal metastasis from lung cancer who was successfully diagnosed using endoscopic ultrasound‐guided fine needle biopsy (EUS‐FNB), with detailed immunohistochemical analysis and systemic imaging findings.

## CASE REPORT

**FIGURE 1:**
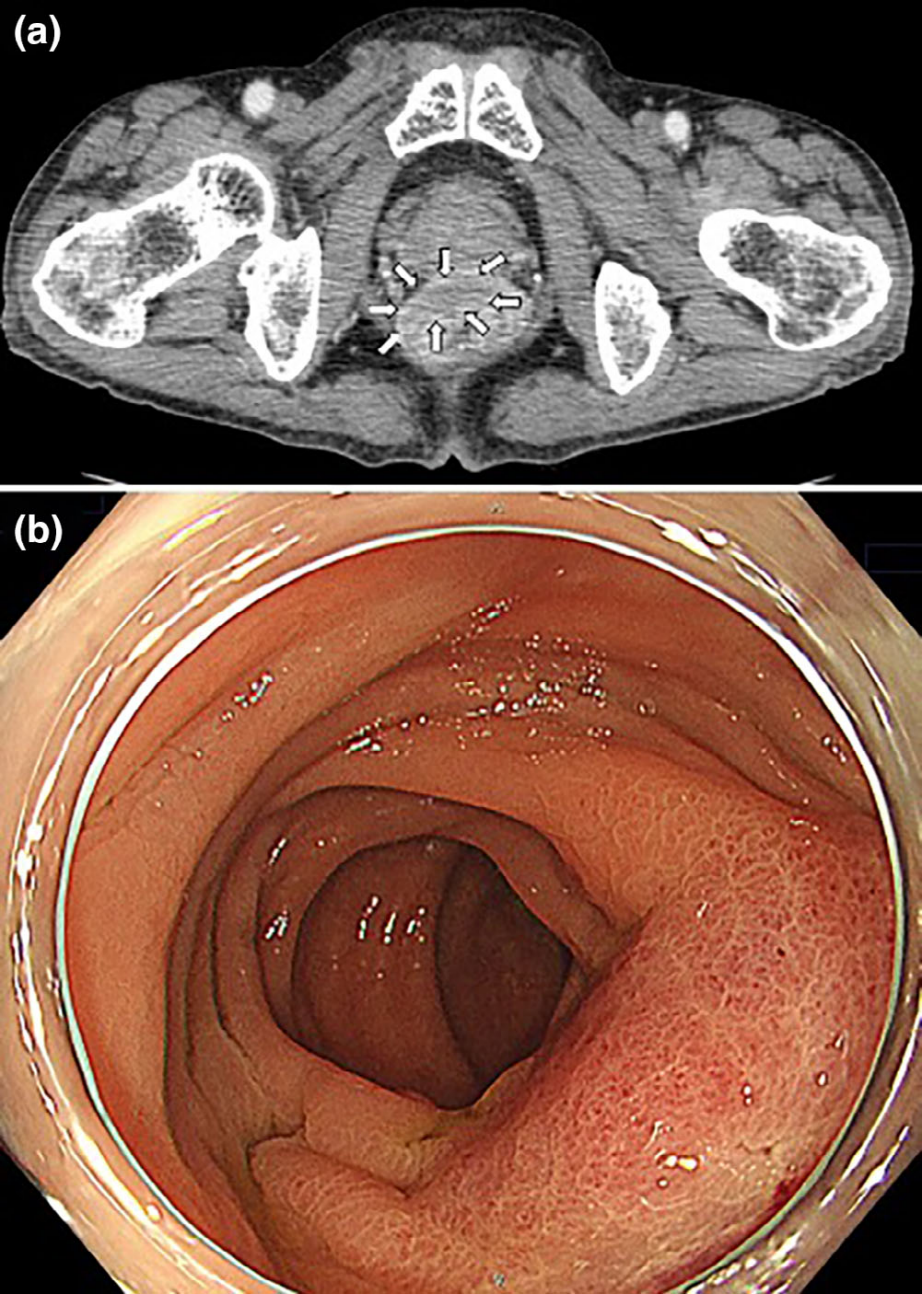
Computed tomography performed on admission. Edematous thickening of the rectal wall with a contrast effect and elevated density of the surrounding fat tissue is shown (a) (arrows). An endoscopic finding. Submucosal tumor‐like protrusion in the Rb lesion of the rectum was observed (b).

**FIGURE 2:**
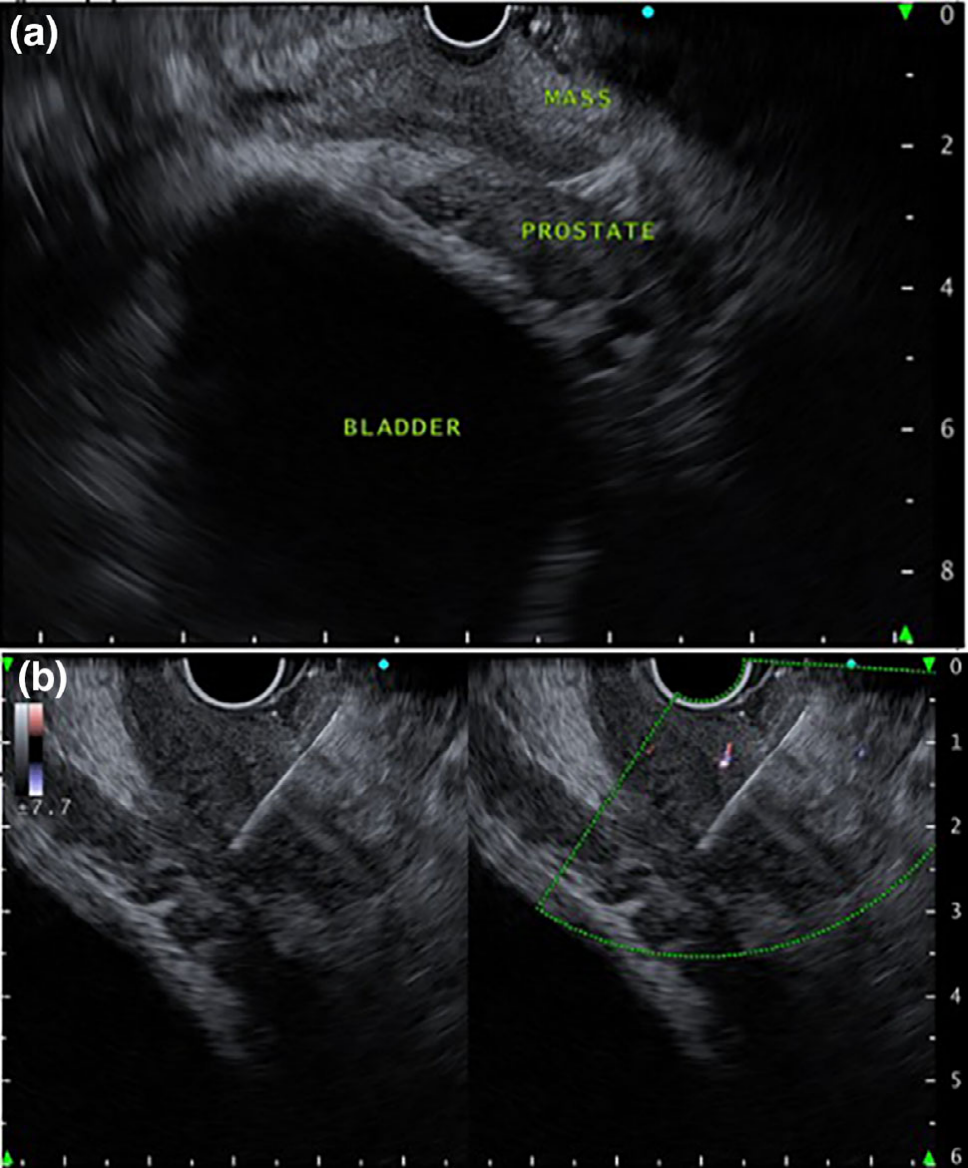
Findings of endoscopic ultrasound of the rectum. A submucosal hypoechoic mass lesion with the possibility of prostatic invasion was observed (a). Endoscopic ultrasound‐guided fine needle biopsy was performed from the lesion (b).

**FIGURE 3:**
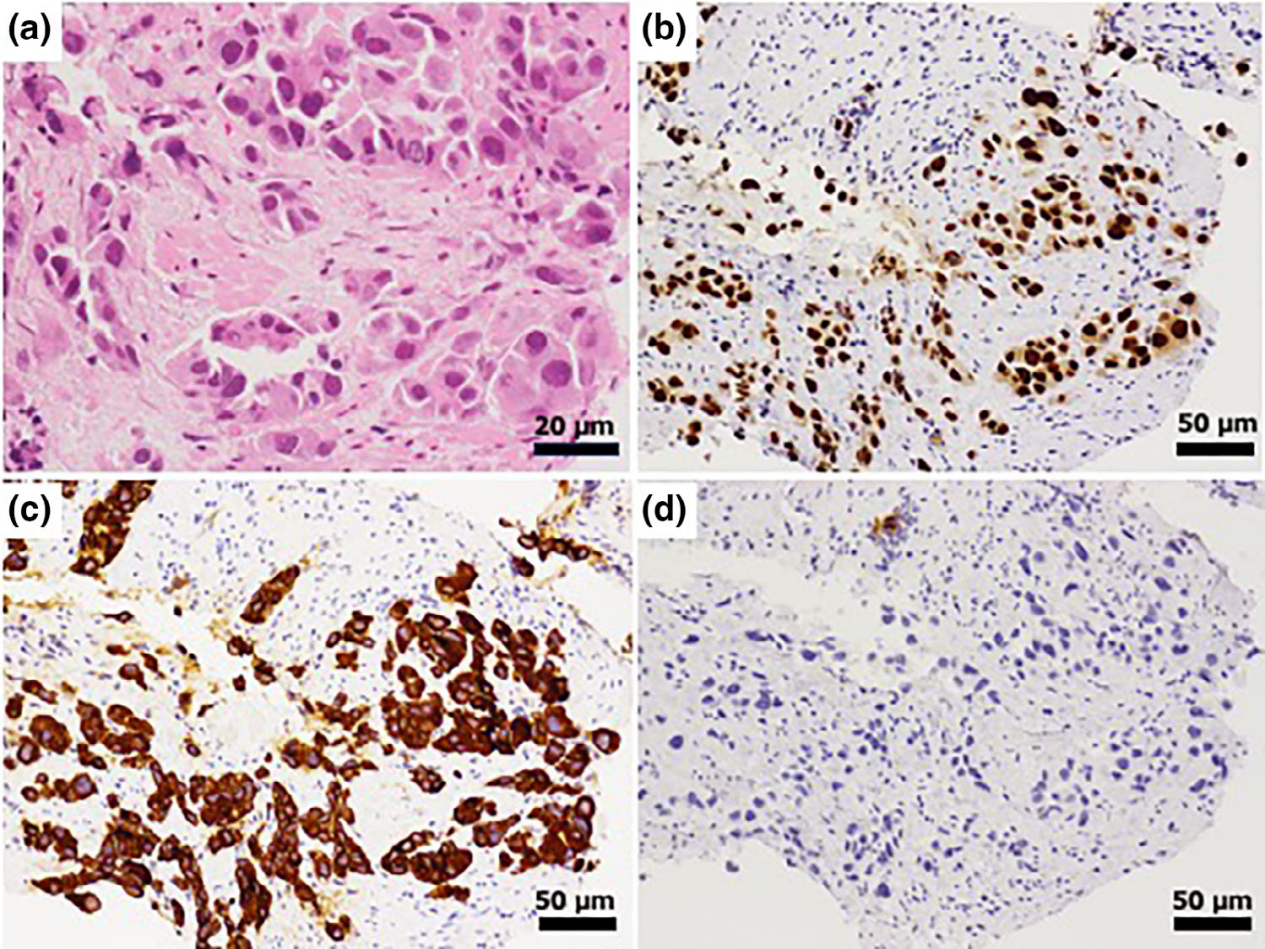
Pathological findings. Hematoxylin‐eosin staining showed a poorly differentiated non‐small cell carcinoma with invasive growth, forming cordate or enhancing foci (a). The carcinoma cells were positive for thyroid transcription factor‐1 (b) and keratin 7 (c), but negative for keratin 20 (d).

**FIGURE 4:**
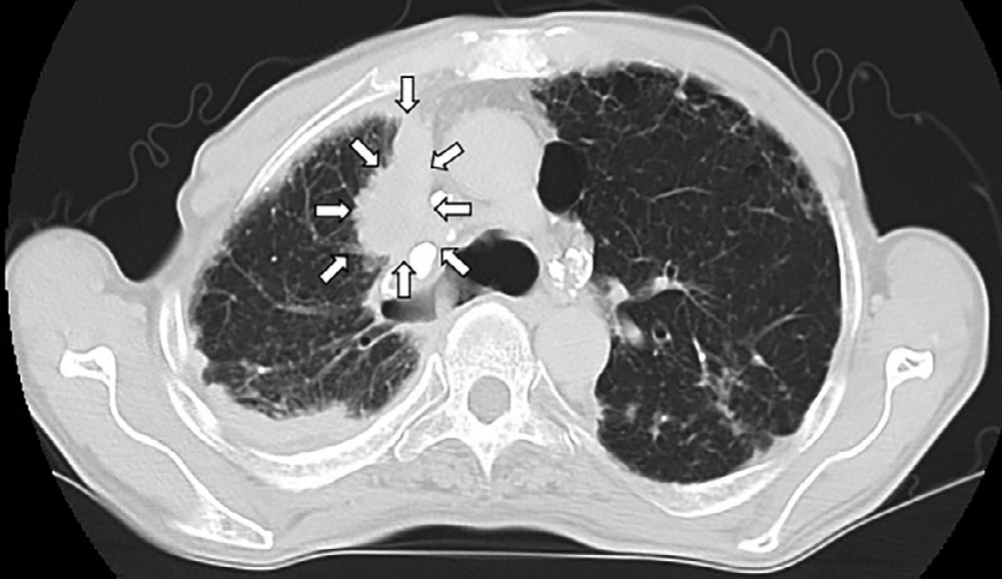
A mass lesion with pleural indentation in the upper lobe of the right lung was observed (arrows).

A 73‐year‐old man with type 2 diabetes mellitus visited our hospital complaining of hyperintestinal peristalsis and diarrhea. The patient had a history of right renal cancer resection and a 53‐year history of smoking 20 cigarettes per day. He had been undergoing regular annual checkups for dust lung disease using computed tomography (CT). One year prior, no obvious neoplastic lesions were observed in the lungs on CT. Except for a high serum carcinoembryonic antigen level (16.1 ng/mL), physical examination and basic laboratory data were within the normal range. Abdominal and pelvic CT scans revealed edematous thickening of the rectal wall with a contrast effect and elevated density of the surrounding fat tissue (Figure 1a). There were no obvious mass lesions in the prostate; however, its boundary with the rectal lesion was indistinct. No other obvious mass lesions were detected on the abdominal and pelvic CT scan. Colonoscopy revealed a submucosal tumor‐like protrusion in the rectum (Rb) without neoplastic epithelial changes (Figure 1b). Although the lumen was slightly constricted, the scope passed through easily, confirming that the entire colon was intact. Forceps biopsies of the lesion revealed no malignant findings. We first suspected he had some submucosal rectal tumors (e.g., gastrointestinal stromal tumor, neuroendocrine tumor/neoplasm, malignant lymphoma) or special histological subtypes of primary colorectal cancer. We conducted EUS‐FNB for the rectal tumor and had planned to perform further investigations depending on the pathological diagnosis. EUS revealed a submucosal hypoechoic mass lesion with a diameter of 26 mm, raising the possibility of prostatic invasion (Figure 2a). Subsequently, EUS‐FNB was performed on the lesion using a linear echoendoscope (GF‐UCT260; Olympus) with a 22‐gauge needle (Trident; Century Medical; Figure 2b). The specimen showed a poorly differentiated adenocarcinoma that had grown invasively, forming cordate or enhanced foci (Figure 3). Alcian blue staining revealed mucus within the sporulation of a few tumor cells (Figure not shown). Immunohistochemical staining showed that the tumor cells were positive for thyroid transcription factor‐1 (TTF‐1; Figure 3b) and keratin 7 (K7; Figure 3c) but negative for keratin 20 (K20; Figure 3d) and prostate‐specific antigen (Figure not shown), which supported the diagnosis of poorly differentiated lung adenocarcinoma (Figure 3b–d). Then, we performed chest CT, and a newly detected mass was observed in the upper lobe of the right lung (Figure 4). Based on the pathological findings and CT images, we diagnosed the patient with rectal metastasis of lung adenocarcinoma. The patient was referred to another specialized respiratory hospital for further examination. Prior to the examination of the primary tumor, the patient developed a complicated rectal obstruction due to tumor growth and underwent a colostomy at the referring hospital. Sputum cytology confirmed the diagnosis of lung adenocarcinoma. Although chemotherapy was planned, his poor general condition necessitated supportive care and he passed away two months after referral.

## DISCUSSION

Metastasis of lung cancer to the gastrointestinal tract is rare, with only 0.5%–1.7% of patients reported to be involved gastrointestinal tract.^1^ Metastasis to the intestinal tract is narrowly defined as tumor embolic metastasis (intramural metastasis), which indicates focal metastasis due to vascular or lymphatic embolization of the tumor within the intestinal wall.^2^ Peritoneal disseminated metastasis and direct invasion are the common patterns of secondary malignancy involving the colon and rectum.^3^

The pathway of intestinal metastasis from lung cancer remains unclear, however, it is generally considered lymphogenous or hematogenous. Hematogenous metastasis leads from local invasion of tumor cells in adjacent tissues to vascular invasion. Then, tumor cells spread to the systemic circulation and proliferate at distant sites. Lymphatic metastasis occurs when tumor cells are deposited intrapulmonary in the lymph vessels surrounding bronchial vessels, spread toward the alveoli, and then metastasize to the regional lymph nodes. Distant metastasis occurs via retrograde metastasis to regional lymph vessels or through other major lymphatic channels. Eventually, tumor cells reach the bloodstream, resulting in secondary hematogenous metastasis^4^. In this case, the enlarged regional lymph nodes were not significant, suggesting hematogenous metastasis.

Colorectal metastases of lung cancer are often exposed tumors and can be diagnosed using endoscopic forceps biopsy.^1^ In the present case, forceps biopsy was not diagnostic, because the tumor was not exposed to the rectal mucosa. Differential diagnoses for rectal submucosal tumors are often difficult only by endoscopic findings. Additional chest CT detected a lung tumor, but it is difficult to differentiate a primary colorectal tumor from a metastatic colorectal tumor. However, as for lung CT findings, lung metastases from colorectal cancer usually present as multiple small round nodules in the peripheral lungs, while primary lung adenocarcinoma shows a single, large, and irregular marginal image with a pleural indentation in the hilar region like the present case. Furthermore, he had a history of dust lung and smoking, which was a high‐risk factor for primary lung cancer. In addition to these imaging findings, pathological evaluation plays a key role in the definitive diagnosis of submucosal tumors.

In immunohistochemistry, only K7/K20 immunohistochemical staining pattern cannot entirely rule out poorly differentiated primary colorectal cancers, although the majority of primary colorectal cancers present K7‐negative/K20‐positive pattern (65.8%) and the minority of it present K7‐positive/K20‐negative pattern (2%) like the present case.^5^ TTF‐1 immunohistochemical staining is useful for differentiating primary lung adenocarcinoma from another adenocarcinoma. It is reported that in differentiating patients with primary lung adenocarcinoma from those with metastatic colorectal adenocarcinoma, the sensitivity and specificity of TTF‐1 are 0.56 and 1.0, respectively^6^. K7 and TTF‐1 are the most sensitive and specific markers for identifying lung adenocarcinoma, while CDX2 and SATB2 are the most sensitive and specific markers for identifying colon cancer.^6^ Although we could not evaluate these markers due to a lack of formalin‐fixed paraffin‐embedded samples, it is very important to perform several immunohistochemical staining of the specimen to differentiate the primary site of the cancer, in addition to referring to clinical course and image findings.

Several studies have investigated the feasibility and safety of EUS‐guided fine‐needle aspiration (EUS‐FNA) for pelvic lesions, but the target lesions have been limited to the rectum or perirectal lesions.^7^ Recently, an EUS scope with an oblique forward optical view was shown to safely insert into the most cephalic bend of the sigmoid colon and puncture the lesions.^8^ On the other hand, the safety of EUS‐FNA for the lower gastrointestinal tract was reported in previous literature which showed that the complication rates were perforation in one (0.2 %) and bleeding in 31 (6.2%) cases out of 502 cases,^9^ but that of EUS‐FNB had not been ever reported. In a large prospective study, the incidence of infectious complications following EUS‐FNA of the lower gastrointestinal tract was approximately 1%, with no differences observed irrespective of the use of prophylactic antibiotics.^9^ In a meta‐analysis of 10 studies comparing the safety of FNB and FNA in 669 patients, it was difficult to evaluate the difference between the two, because there were no reports describing adverse events with a statistically sufficient number. The most frequent adverse event was minor hemorrhage with only six cases in total (<1%)^9^. Furthermore, the European Society of Gastrointestinal Endoscopy recommends EUS‐FNB or mucosal incision‐assisted biopsy equally for the tissue diagnosis of subepithelial lesions larger than 20 mm in size.^10^ If puncture of the lesion is possible, the FNB needle is more likely to collect an appropriate specimen; however, the FNA needle can be selected according to the size and shape of the lesion. As no reports have described trans‐colorectal EUS‐guided tissue acquisition using an FNB needle as in the present case, further investigation and accumulation of similar cases are necessary.

In conclusion, we report a case of rectal metastasis from lung cancer that was successfully diagnosed using EUS‐FNB and detailed immunohistochemical analysis. In cases of a non‐exposed rectal tumor, EUS‐FNB and systemic evaluation using CT can be informative in cases of a non‐exposed rectal tumor.

## CONFLICT OF INTEREST STATEMENT

None.

## ETHICS STATEMENT

N/A

## PATIENT CONSENT STATEMENT

Informed consent was obtained from the patient to be included in this report.

## CLINICAL TRIAL REGISTRATION

N/A
